# Gallbladder Bilharziasis

**DOI:** 10.1155/1996/76282

**Published:** 1996

**Authors:** Mohamad Abid Bakhotmah

**Affiliations:** Department of Surgery King Abdulaziz University Hospital P.O. Box 6615 Jeddah 21452 Saudi Arabia

## Abstract

A 30 years old man presented with symptoms of Bilharziasis. Ultrasound showed gallstones in the gallbladder which was removed laproscopically, heavy bilharzial infection was detected in the gallbladder tissue. The first case in Saudi Arabia is reported. A Review of the world literatures since 1966 about the subject is presented and different aspects of the gallbladder schistosomiasis are discussed.


*HPB Surgery*, 1996, Vol.9, pp.175–177
Reprints available directly from the publisher
Photocopying permitted by license only

© 1996 OPA (Overseas Publishers Association)
Amsterdam B.V. Published in The Netherlands
by Harwood Academic Publishers GmbH
Printed in Malaysia

### CASE REPORT
### Gallbladder Bilharziasis

MOHAMAD ABID BAKHOTMAH

Assistant Professor and Consultant Hepatobiliary Surgeon. Department of Surgery, King Abdulaziz University Hospital, P.O. Box 6615, Jeddah 21452, Saudi Arabia

*(Received 14 March 1995)*

A 30 years old man presented with symptoms of Bilharziasis. Ultrasound showed gallstones in the gallbladder which was removed laproscopically, heavy bilharzial infection was detected in the gallbladder tissue. The first case in Saudi Arabia is reported. A Review of the world literatures since 1966 about the subject is presented and different aspects of the gallbladder schistosomiasis are discussed.

KEYWORDS: Gallbladder parasite parasitology bilharziasis schistosomiasis

### INTRODUCTION

Schistosomiasis (Bilharziasis) cause a major health problem in many parts of the developing world. The most likely-but not definite-site for adult worm depends on the species; (S. haematobium in vesical veins, S. Mansoni in superior mesenteric veins. S. Japonicum & S. Mekongi in the inferior mesenteric vein) but worms can live in any of the location and mixed infection are also common. Eggs are deposited in the venules of the corresponding location of the adult worm and find their way to urine or faecies to be excreted, however certain eggs are either trapped on the way or even lose their way and settle in ectopic locations; one of the extremely rare location is the gallbladder, we report a case of schistosomal (bilharzial) cholecystitis. Using a "Medline" search between 1966 and December 1994, there were no reported case or study concerning gallbladder bilharziasis; this is the first case of gallbladder bilharziasis with gallstones causing chronic cholecystitis to be reported from Saudi Arabia.

*Correspondence to*: Dr. M. A. Bakhotmah, MBBS, FRCS(E) Assistant Professor and Consultant Hepatobiliary Surgeon, Department of Surgery, King Abdulaziz University Hospital, P.O. Box 6615, Jeddah 21452, Saudi Arabia,Tel. No.: 02-6952845 Fax No.: 02-6952885

### CASE REPORT

A 30 year old man presented to the general clinic with haematuria and right upper quadrant pain, Urine analysis showed heavy infection with S. haematobium and ultrasonography showed a solitery gallbladder stone. Other investigations were normal. Although the right upper quadrant pain was not typical of biliary pain there was no renal stone to explain it; Laproscopic cholecystectomy was performed after treating his schistosomiasis by Braziguantil. The Postoperative period was uneventful. Histology of the gallbladder showed chronic cholecystitis and schistosomal ova were seen in the wall of the gallbladder.

### PATHOLOGY

Grossly, the gallbladder contains one small stone and the wall was thickened. Microscopically (Fig.1) the mucosa is lined by columnar epithelium and shows lymphocytic infiltrate of the lamina propria with occasional eosinophils. The muscle coat is hypertrophied, fibrosed and infiltrated by lymphocytic cells. Numerous ova of schistosoma haematobium with terminal spine (inset 1) and mansonai with lateral spine (inset 2) are present in the gallbladder wall. There was focal granulomatous reaction found elsewhere. The appearances are that of chronic cholecystitis with schistosomal infection.

### DISCUSSION

Schistosomal eggs which pass to the lumen of the gut or urinary bladder evoke no host reaction, those eggs which do not move fast or move in the wrong direction (as those found in the liver) are soon surrounded by inflammatory cells and granulomatous and fibrotic reactions ensure[1]. The pathogenesis of chronic schistosomiasis is almost exclusively explained by this granulomatous reaction. Although liver is one of the most commonly affected organs -leading to liver fibrosis and portal hypertension the biliary system is seldom affected by complications of schistosomiasis. No tissue or organ is immune from wide dissemination of schistosomal eggs. – Ectopic granulomatous lesions may be found in perigenital tissue, spermatic cord, epididymis, testes, uterine cervix, ovary, meninges, spinal cord, brain tissue, stomach, oesophagus and pancreas[1–3]. As ascariasis[5] and cryptosporidiosis[6]; schistosomal ova were found in mucosa, submucosa, fibrovascular coat or even free in the gallbladder content[7]; but still, schistosomal granulomas of the gallbladder causing cholecystitis is very rare[4]. In an important study of the distribution of the schistosomal lesions in various organs among 1220 autopsies 2.5% of cases was affected by gallbladder bilharziasis compared to 35% and 5% of the liver and pancreas respectively[8]. The explanation of this variation in incidence among organs is probably due to variation of the richness of venous drainage, the more veins available in the part the more will be the chance for the female worms to get in and lay ova[8]; mixed infection of the gallbladder has never been addressed before but in our case a mixed infection was documented. The

**Figure 1** Section of the gallbladder showing lymphocytic infiltrate and Schistosoma ova, S. haematobium (inset 1) and S. mansoni: (inset 2). The lumen contains bile pigment (b). (See color plate I)

thickened wall and the impaired gallbladder contraction after the fatty meals in patient with bilharzial hepatosplenomegaly[8] lead to the suspicion that they may be more prone to develop gallstones, however this is not proved yet.

Schistosomal ova can form a nidus for the development of renal stones[4]. Neither we nor others were able to detect any schistosomal ovum in the gallstones retrieved from patients. Liver biopsy was not done because it was not necessary as liver function tests and laproscopic appearance of the liver was normal.

### REFERENCE

1. Mansour-Bahr P.E.C., Apted F.I.S., Schistosomiasis in: Mason-Bahir P.E.C., Apted F.I.C eds., (1984) The Manson's Tropical Medicine, Eighteenth Edition, London, Bailliere Tindal Publishing Co. 207–228.
2. Strickland G.I., Abdel-wahab. M.F., (1991) Schistosomiasis in: Strickland G.T. ed., Hunter's Tropical Medicine, Seventh Edition, Philadelphia, W.B. Saunders Publishing Co;781–808.
3. Mahmoud A.F., Abdelwahab, M.F., (1990) Schistosomiasis in: Warren KS, Mahmoud A.F. eds, Tropical and Geographical Medicine, Second Edition, New York, McGran-Hill Information Services Publishing Co., 458–473.
4. Abdelwahab M.F., (1982) Schistosomiasis in Egypt, CRC press, ISBN 0-8493-6220-2, 132–138.
5. Gracia L.S., Current W.L., (1989) Cryptospordiosis Clinical Features and Diagnosis, *Crrt-Rev-Clin Lab-Sci*, **2** (6): 439–60.
6. Khuroo, M.S., Zorgor S.A., Mahajan R., (1990 June 23) Hepatobiliary and Pancreatic Ascariasis in India, *Lancet*, , **335** (8704): 1503–6.
7. Massoud A.A., Farrag S.A, Makarem S.S, *et al.*, (1988) The gallbladder as a cause of dyspepsia in Egyptian schistosomal hepatosplenomegalic patients, *Journal of the Egyptian Society of Parasitology*; **18** (1): 133–140.
8. El-Rooby A., (1976) The gastroentrology of schistosomiasis, compiled review of schistosomiasis in Egypt, pp 137–143.

